# Pharmacists in general practice: a qualitative interview case study of stakeholders’ experiences in a West London GP federation

**DOI:** 10.1186/s12913-018-3056-3

**Published:** 2018-04-02

**Authors:** Kath Ryan, Nilesh Patel, Wing Man Lau, Hamza Abu-Elmagd, Graham Stretch, Helen Pinney

**Affiliations:** 1 School of Pharmacy, University of Reading, Whiteknights Campus, PO Box 226, Reading, RG6 6AP UK; 2 Ealing GP Federation, 179C Bilton Road, Perivale, Greenford, Middlesex, UB6 7HQ UK; 3 School of Pharmacy, Faculty of Medical Sciences, Newcastle University, Newcastle upon Tyne, NE1 7RU UK

**Keywords:** Pharmacists, General practice, Roles, Education, Communication, Trust, Patients’ awareness, UK

## Abstract

**Background:**

Increased patient demand for healthcare services coupled with a shortage of general practitioners necessitates changes in professional roles and service delivery. In 2016, NHS England began a 3-year- pilot study of pharmacists in general practice, however, this is not an entirely new initiative. There is limited, current, evidence-based, UK research to inform the pilot so studies of pre-existing services must suffice until findings from a formal national evaluation are available.

**Methods:**

The aim of this exploratory, descriptive interview study was to explore the experiences of stakeholders in eight general practices in the Ealing GP Federation, West London, where pharmacy services have been provided for several years. Forty-seven participants, including pharmacy team members (pre-registration and clinical pharmacists, independent prescribers and pharmacy technicians), general practitioners, patients, practice managers, practice nurses and receptionists took part in semi-structured, audio-recorded qualitative interviews which were transcribed verbatim, coded and analysed thematically to extract the issues raised by participants and the practicalities of providing pharmacy services in general practice.

**Results:**

Findings are reported under the themes of Complementarity (incorporating roles, skills, education and workloads); Integration (incorporating relationships, trust and communication) and Practicalities (incorporating location and space, access, and costs). Participants reported the need for time to develop and understand the various roles, develop communication processes and build inter-professional trust. Once these were established, however, experiences were positive and included decreased workloads, increased patient safety, improved job satisfaction, improved patient relationships, and enhanced cost savings. Areas for improvement included patients’ awareness of services; pharmacists’ training; and regular, onsite access for practice staff to the pharmacy team.

**Conclusions:**

Recommendations are made for the development of clear role definitions, identification of training needs, dedication of time for team building, production of educational materials for practice staff members and patients, and provision of on-site, full-time pharmacy services. Future work should focus on evaluation of various models of employing pharmacy teams in general practice; integration of pharmacists and pharmacy technicians into multidisciplinary general practice teams; relationships between local community pharmacy and general practice personnel; and patients’ service and information needs. A formal national evaluation of the pilot scheme is overdue.

**Electronic supplementary material:**

The online version of this article (10.1186/s12913-018-3056-3) contains supplementary material, which is available to authorized users.

## Background

Currently, there is a nationwide shortage of 8000 General Practitioners (GPs) across the UK [1]. In contrast, there is a surplus of registered pharmacists, with the excess number, according to The Centre for Workforce Intelligence, expected to be 11,000–19,000 by 2040 [1] if roles remain unchanged. As a result, in 2015 the Royal Pharmaceutical Society (RPS), in collaboration with the Royal College of General Practitioners (RCGP), proposed further integration of pharmacists into general practices outlining the various benefits that pharmacists could provide. In 2015, NHS England announced a 3-year pilot at a cost of £31 million to test the role of pharmacists working in general practices, which resulted in 490 pharmacists employed across approximately 650 practices. In early 2017 further funds (£122 million) were committed to support an extra 1500 positions by 2020/21 [2].

Having a pharmacist in a general practice team is not new [3]. Because of recent changes in, and rapid development of, the role of pharmacists, there is limited current UK research that could inform the pilot [4, 5]. Alongside impact metrics, work is needed on pre-existing collaborations. Early pilot sites can provide preliminary insights into the practicalities, benefits and barriers of employing pharmacists in general practice, multidisciplinary team development, and stakeholder experiences of service provision. One pre-existing collaboration, now a pilot site, is in the Ealing GP Federation in West London. In 2016, eight practices serving approximately 70,000 patients had already been contracting a private company to provide pharmacy-related services for approximately 3 years prior to the pilot. The pharmacy team, at that time, was comprised of a prescribing lead pharmacist (manager), lead pharmacy technician, field-based clinical diploma pharmacist, six prescribing pharmacists, six technicians, a pre-registration trainee pharmacist and prescription delivery drivers. They provided a variety of services, including face-to-face medication reviews; long term condition, repeat prescription and medication management; triage; and acute care. Some services were provided in the general practice and some in domiciliary settings, including surrounding residential aged-care and nursing homes. The Ealing GP Federation model of integration of pharmacy services into general practice is probably unique in employing pharmacy technicians but no work has been done to determine the variety of integrating models in existence across the UK. Because of its pre-existing nature, this model provides a timely case study to give insight into how integration might evolve given time.

International studies, mostly in Australia, New Zealand and Canada, have primarily focussed on the experiences of GPs, pharmacists and patients [4–15] and have shown positive GP responses towards the integration of pharmacists into general practices. GPs recognised that having a practice-based pharmacist decreased their workload and allowed them to focus on their diagnostic and prescribing roles, while pharmacists provided expert medication advice and patient counselling [4, 10, 14]. Potential barriers to integrating pharmacists into general practices were cited as a lack of readiness in patients to accept them; the perceptions of an already established and rigid hierarchy amongst existing staff within the general practice; and the limited time that pharmacists spent there [8, 10, 11, 13]. Gaps in the literature include the experiences of other stakeholders involved in the patient’s care. Therefore, in this exploratory study we interviewed six stakeholder groups aligned with the Ealing GP Federation (pharmacy team members, GPs, patients, practice managers, receptionists and nurses) to answer the question: What are the experiences of the various stakeholders in a group of general practices currently employing pharmacists?

In an attempt to inform future development of new pilot sites, in this paper we provide a descriptive report of stakeholders’ experiences and outline their views on the practicalities of setting up and maintaining pharmacy services in general practice.

## Methods

A pair of researchers, from a group of 12 final year Master of Pharmacy students was assigned to each one of the six stakeholder groups. The aim was to interview up to 12 people, unknown to the researchers, from each group to capture a wide range of experiences. The researchers received qualitative interview training from supervisors with qualitative expertise and were given detailed feedback on their early interviews. In-depth, semi-structured, face-to-face interviews were conducted during Jan-Mar 2016.

All staff members at the practices were contacted by email by the lead pharmacy technician. They were provided with a copy of the invitation letter, participant information sheet and consent form and asked to notify the lead pharmacy technician of their willingness to participate. A time was then arranged for the interview. One follow-up email was sent approximately 2 weeks after the first.

Eligible patients (aged 18 or above, competent to give informed consent and English language speakers) were identified by the lead pharmacy technician at the time of booking a medication review appointment with the lead pharmacist. Patients were provided with an invitation letter, participant information sheet and consent form and invited to take part. Once consent was obtained the interview was scheduled directly after the patient’s medication review with the pharmacist.

All participants who agreed to be interviewed gave their written, informed consent before participating in the study. Semi-structured, in-depth interview guides were developed by the research team based on the known nature of the participants’ roles and findings from the scant published literature from overseas (Additional file 1). Questions were designed to allow participants to talk in a conversational manner about their experiences in their own terms. Open-ended questions, such as “What are your roles and responsibilities in this GP surgery?”, “Tell me about your experiences of having/being a pharmacist working in this GP surgery” and “How have pharmacists in this GP surgery affected healthcare provision?” were followed up with prompts, for example about experiences, workloads, quality of relationships, feelings, barriers and facilitators to elicit more detailed answers. Interviews were largely guided by the participants themselves and they chose what to discuss and how much to disclose. All interviews took place in a private area of the general practices.

The interviews were audio-recorded and given a code according to their stakeholder group and interview number (for example GP1 = general practitioner 1; M1 = manager 1; N1 = nurse 1; PA1 = patient 1; P1 = pharmacist 1; IP1 = independent pharmacist 1; CP1 = clinical pharmacist 1; PT = pharmacy technician; R1 = receptionist 1 etc). The recordings were downloaded to password protected computers as soon as practicable, after which the audio recorders were cleared. Interviews were transcribed verbatim with all information anonymised, checked by each pair of researchers, and stored on a university password protected server. The first interview transcript was reviewed by the lead researchers with each pair who were encouraged to be reflexive about their techniques, and feedback was provided before further interviews were scheduled.

For this paper, data from each pair of student researchers was pooled by another researcher (HAE), managed using NVivo 11 (QSR International), coded inductively from the interview transcripts and analysed using interpretive thematic analysis [16, 17] and constant comparison [18]. The research team met regularly throughout the data analysis process to reduce the possibility of researcher bias. Potential categories were identified, discussed by the authors and confirmed by detailed re-reading of the transcripts. Further refinement and reduction occurred until a final list of categories was obtained and agreed by all the lead researchers. The themes were then finalized by collapsing categories under descriptive titles that reflected the content of the data and addressed the aim of the study. Illustrative quotes, that represented the range of stakeholders, their experiences of pharmacists in general practice, the issues they raised and the analytical points being made, were selected for reporting and appear in the text in italics.

## Results

Forty-seven stakeholders took part in interviews lasting 15–45 min. Table 1 shows the number interviewed and the total number of potential interviewees in each stakeholder group together with basic demographics. To guarantee anonymity, further demographic details could not be included. It can be seen that reasonable numbers of potential general practice staff participants were interviewed which provides credibility that the widest possible range of experiences and opinions was obtained. Only nine patients were available for interview in the time frame allocated.

**Table 1:** Characteristics of stakeholder participants (n = no. of interviewees/total pool)

Demographic	Stakeholder group						
	GP (*n* = 7/50)	Nurse (*n* = 6/18)	Manager (*n* = 8/9)	Patient (*n* = 9)	Pharmacist (*n* = 5/8)^a^	Pharmacy technician (*n* = 4/6)	Receptionist (*n* = 8/43)
Age (yrs)							
< 25					1		1
25–34	2				2	2	1
35–44	3				1	2	1
45–54		1					
55–65				1	1		
> 65	1			4			1
Missing	1	5	8	5			4
Time at practice (yrs)							
< 5	3	3	8	1	4	4	5
5–10	2				1		3
11–15	1	1		2			
15–20		1		1			
> 20	1	1		1^b^			

^a^Pharmacist group includes one pre-registration pharmacist

^b^Unable to obtain the time at the practice for 4 patients

**Fig. 1:**
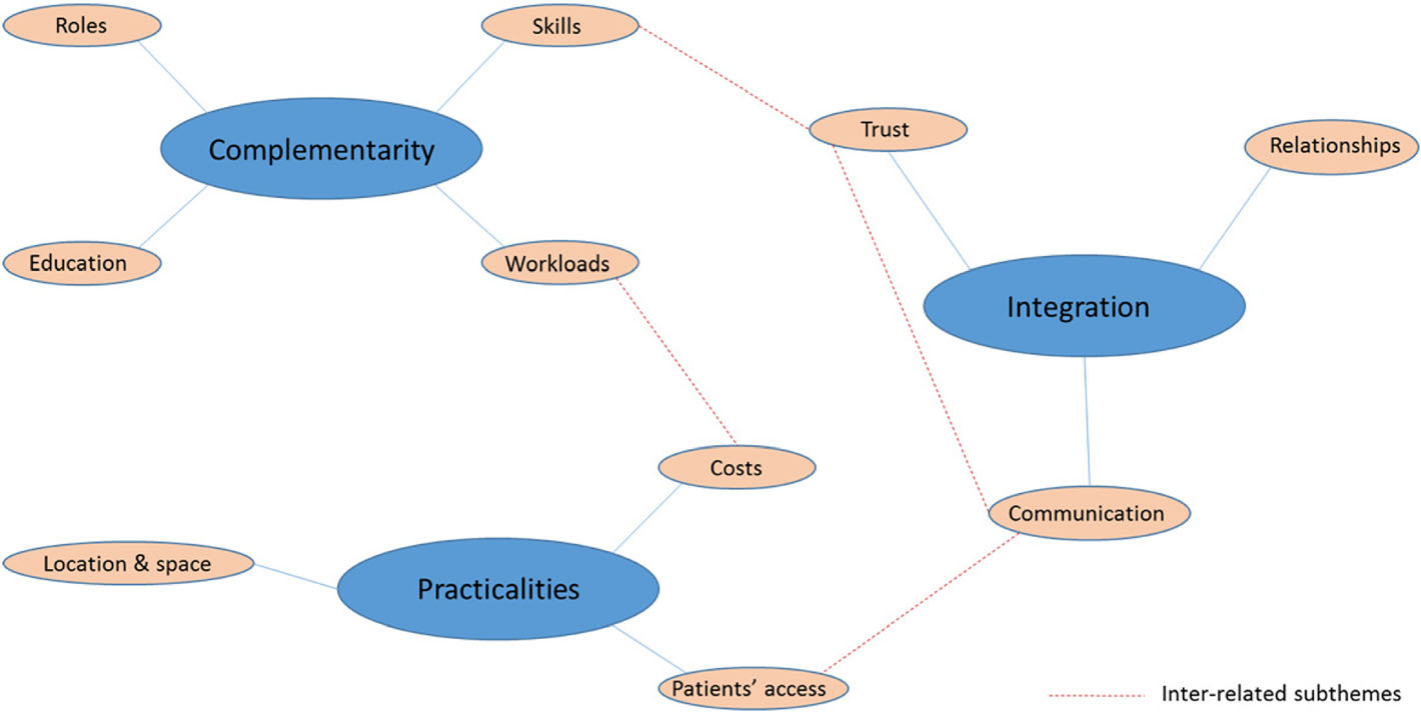
Analytical themes and sub-themes

Figure 1 illustrates the three main themes and (inter-related) subthemes of practical significance derived from the data that are presented here: Complementarity (incorporating roles, skills, education and workloads), Integration (incorporating relationships, trust and communication) and Practicalities (incorporating location and space, access and costs).

### Complementarity

Complementarity is defined, by the Oxford Dictionary of English, as a relationship or situation in which two or more different things improve or emphasize each other’s qualities. Participants discussed the complementary nature of their roles and skills and the contribution, such as decreased workloads, that pharmacists made to the multi-disciplinary team. They also talked about the need for appropriate educational training and a clear understanding of professional competencies.

#### Roles

Roles identified for the pharmacy team included face-to-face appointments with patients to manage long term conditions, polypharmacy, medicines optimisation and patient monitoring; spirometry clinics; treatment of common ailments; telephone triage of medicines enquiries; management of repeat prescriptions; reconciliation of discharge summaries; Quality and Outcomes Framework (QoF) monitoring and reporting; and the completion of prescribing audits. The pharmacy team was supervised by an experienced lead pharmacist and pharmacy technician. All pharmacists had a patient facing role and those who had completed independent prescribing shared responsibility for authorising prescriptions. All practitioners were required to act within their respective competency. Clinics were booked up to a month in advance by receptionists, GPs and nurses. Pharmacists received mentorship by the lead pharmacist and a named GP. Technicians were trained and supervised by the lead pharmacist and pharmacy technician. The processing of prescriptions was done both on site and remotely using IT solutions (bespoke electronic systems and support) including N3 (national broadband network for the NHS in England) connected secure laptops with access to SystmOne (the medical records software), on-line ordering for patients, scanning of paper orders and letters and NHS net secure email. Use of electronic transmission of prescriptions to community pharmacies wherever possible and secure transport of paperwork by drivers was also deployed.

*They do medication reviews… they do rounds in nursing homes where they check all medication is being used appropriately… medication control and health promotion, lifestyle advice all those sort of things, could definitely be done by the pharmacist. So there is definitely more roles for pharmacists in general practice (M2).*

The arguably unique feature of the Ealing GP Federation model of pharmacy services provision is the novel management of repeat prescriptions utilising the expertise of pharmacy technicians which was described as *a light bulb moment (GP2).* There was a strong belief amongst receptionists and managers that their previous involvement in repeat prescribing was beyond their skillset and participants reported being more *confident* (M5) in a process that was *much safer* (R3) under the supervision of a pharmacist. Repeat prescription requests, for example, were referred by the receptionists to the pharmacy team and were processed and issued according to a “48-h turnaround” protocol. The pharmacy technicians referenced the prescription request against the protocol and patient medical records and, if set criteria (such as an appropriate blood test, medication review and compliance with local formulary) were met, the prescriptions were generated for a signature, most commonly from the prescribing pharmacist or, where appropriate for items requiring medical input, the patient’s GP. Medication reviews were directed by the technicians to the most appropriate healthcare professional, such as a GP, nurse or practice pharmacist. Alternatively, patients had the option to directly make an appointment to see the pharmacist if they wished to discuss medication-related concerns.

Many members of staff reported increased understanding of the role of pharmacists in general practice although awareness remained limited for some receptionists, nurses, managers and patients.

*It took us a while to get the nurses to realise that when we refer to the pharmacy team, they were different from their chemist shop and that caused confusion at the beginning (M4).*

*Let’s say the situation is whether to continue a medication the patient is on or specifically when patients complain about medication. A pharmacist will not be able to answer that. It’s more clinical so I’d refer to see a GP… I don’t think the practice is that desperate for a pharmacist (N6).*

It was suggested that to facilitate effective working between pharmacists and other practitioners *you’d have to have regular meetings to help understand what each other’s roles are (N1).* All staff members identified the pharmacy team’s added contribution to the practice through educational talks.

*We had a meeting with the prescribing advisor which the pharmacy team came to, that was quite a good discussion, particularly having a pharmacist there who really knows their stuff… It was a different conversation than previous prescribing meetings… a lot more focused on particular medication and latest evidence (M2).*

Pharmacists and technicians described increased job satisfaction derived from their extended roles, including involvement in patient care, greater use of their skills and increased responsibility.

*It really diversifies my role, so I don't feel like I'm just stuck in the dispensary. I feel like I'm empowered to help patients to a greater degree (IP2).*

*This is the best job I've had, because… I see the whole patient history, I see hospital letters, I see pathology, I see the whole picture. Whereas in the community pharmacy I could only see what they had come in with, the medicines (IP1).*

Future roles were discussed by GPs and managers. They identified the potential for pharmacists’ increased involvement in the management of long-term conditions and the establishment of a minor ailments clinic. One manager identified a potential future role for a *pharmacy technician healthcare assistant with knowledge about medication and... [the ability to] take blood pressure, weight, height, all those kind of things (M2).*

#### Skills

Members of every stakeholder group thought that pharmacists had a strong knowledge of medications, citing many instances in which they had used the pharmacy team for information and advice. GPs and nurses described the pharmacists’ knowledge of medications as superior to their own.

*Their knowledge is way over and above anything we know about drug interactions. (The pharmacist) can just look at a list of medications “They shouldn’t be on that, they should be on this” and he’s very, very good at picking up on the subtleties of medication interactions and the NICE guidance (GP6).*

Two patients (PA3 & PA5) believed that the pharmacist *knew more about medication than the doctors (PA3)*. Several said that they had not previously thought to ask about their medicines and that the information they received from the pharmacist was beneficial and important, concluding that the pharmacist was the most appropriate person to speak to for medication related queries.

*I don’t even need to see a doctor… (The pharmacist is) so good she explains everything… not that I mind one or the other but… I can see that the pharmacist for me is all I need (PA7).*

GPs acknowledged the varied skills that they felt the pharmacy team possessed outside their defined roles, citing the pharmacists’ ability to counsel beyond pharmacological management including holistic health and lifestyle advice, their ability to apply their knowledge in a practical setting and their methodical approach.

*(The pharmacist) has an interest in elderly care so he doesn’t always go to pharmacological management. He would be able to advise outside that as well and… he understands intrinsically the importance of not just rushing into polypharmacy in elderly patients…. It is quite holistic actually and it’s quite easy for a lot of doctors to fall into that trap. So he will just say “Why not think about this or think about that”. I found that quite useful (GP3).*

Participants also described increased efficiency within the general practice in dealing with the repeat prescribing process with one manager (M1) reporting a reduction in medication-related complaints. There was concern, however, from two GPs and a nurse, but without evidence, that lack of involvement might lead to them (or younger GPs) *losing confidence (GP7)* and becoming *deskilled (GP4)*, through non-engagement with (repeat) prescribing and loss of continuity of patient care, resulting in reduced knowledge of patients’ current medications. This, however, was not a universal sentiment.

*Some GPs are worried about getting deskilled about repeat prescribing, I don’t think it’s any great loss, the patients are safer as such. I am not bothered as it’s their safety, patient safety is better (GP3).*

#### Education

Some pharmacists expressed the need for postgraduate training, believing that the knowledge they gained from a pharmacy degree and pre-registration year was insufficient to work effectively in general practice.

*It's not simply coming out of university and working in a GP surgery - that would be very uncommon, you would need further training, either through your pre-reg or after you've done your pre-reg, a diploma or something (IP2).*

Some pharmacy technicians also expressed a desire for further training feeling that they were initially underequipped for their role.

Although pharmacists readily identified the boundaries of their expertise, one GP repeatedly expressed concerns that a pharmacist might act outside of their professional competencies.

*The major risk for me, if you get some pharmacists working in practices in a clinical role, is them not knowing what they don’t know (GP2).*

#### Workloads

All GPs stated that the introduction of a pharmacy team reduced their involvement in medication management, facilitated more appropriate use of their skills, and allowed them to increase their patient-facing activity leading to improved relationships.

*It’s brilliant having (a pharmacist)… they are a fantastic resource but they are also significantly reducing our workload (GP6).*

*You are going to be seeing more patients during the day, you get to know your patients better rather than just dealing with looking at the computer doing medications. When you’re doing medication reviews you get a lot of patients that are on six, seven, ten plus medications and arranging that takes a bit of time so you are not spending that with your patient. You are not giving your full concentration and it does affect your patient-doctor relationship (GP7).*

*(Repeat prescriptions are) a hugely time consuming thing, I think doctors were spending a good half hour, 40 minutes [a day] on prescriptions. Equally a lot of our on-call sessions would be dealing with script queries from patients, them saying they had ordered something and it hadn’t arrived that kind of stuff. So all of that has been skimmed off the top of our workload basically, so it is quite a big impact on staff and doctors as well (GP5).*

Changes in workloads were not limited to GPs with receptionists, managers and nurses also reporting a reduction. One receptionist (R6) revealed that, prior to the pharmacy teams’ introduction, they were tempted to leave due to their high workload but now believes that this has allowed them to increase their focus on individual patient needs rather than medication queries.

Although occasionally there were concerns raised over a high workload, the pharmacy team’s experiences were overwhelmingly positive.

*Often the demand and the expectations of what one person can actually do in the time allocations (are too high) (IP2).*

One pharmacy technician (PT1), however, thought that the receptionists unnecessarily relied upon them referring queries that they, the receptionists, could have dealt with themselves.

### Integration

Introduction of a pharmacy team into general practices incurred some initial hurdles around building trusting relationships both within the practice and with surrounding community pharmacies but with time and good communication this group of practices was able to overcome most of the barriers.

#### Relationships

Most participants reported positive and healthy professional and personal relationships between the pharmacy team and the rest of the practice staff with little hesitation in approaching the team with queries and vice versa.

*My relationships with the doctors are very good. I can knock (on the door of) any doctor at any time and discuss a case. For example, if I'm not happy with a particular medication or I feel not comfortable in signing the prescription I want to speak to the last doctor who has actually seen the patient (IP3).*

*It’s nice, everyone helps each other, things are done on time. If they (the pharmacy team) make a mistake, they fix it, so there are never really any issues. Best thing they’ve done was get them here, I think (laughs) (R6).*

*I think some of the doctors probably have a close networking relationship (with the pharmacists), the partners for example. We [GP partners] initially met them and employed them so for example today I had a patient call up, and I sent a quick instant message to [Pharmacy technician] who was upstairs and asked him “What would you suggest as an alternative?” and then 2 minutes later the patient could have their prescription, all done and dusted. Equally with [Pharmacist] who is obviously in charge of the whole thing. He is very happy for us to drop him an email and usually replies very quickly if you need advice or anything like that, and we all get on very well on a personal level too. He comes to our Christmas parties. So yeah we’ve found that there’s also a lot of informal support as well [as] with the actual day to day business of regenerating prescriptions for us and things (GP5).*

Pharmacy technicians and receptionists reported a close working relationship, however, two receptionists felt that some members of the pharmacy team were not always cooperative, being reluctant to take calls coming through reception.

*I get along with the pharmacy team generally but when it comes down to having to crack down on things, it is a bit like, “I know more than you”, if you get what I mean from their end (R8).*

*They only wanted to issue and sign, which is understandable. They didn’t want to take all the calls, only the calls that were necessary. That was the hardest part at the beginning but now they are fine (R6).*

Participants’ opinions on the relationship with local community pharmacies were mixed. One GP suggested that *the trust level was low (GP6)* while one nurse described the local pharmacist as *really helpful (N1)*. Examples were provided of the general practice pharmacy team liaising with local community pharmacies. One GP believed that the presence of the team could *improve the relationship with community pharmacists* and that the practice pharmacist *could organise things that would have taken (the GP) hours (GP3)*. Members of the pharmacy team were also of the opinion that their presence in the general practice had bridged the gap between the two pharmacy settings.

Participants mentioned that the pharmacy team were involved in challenging local pharmacies on unethical practices, including inappropriate community pharmacy repeat prescription requests (N2 & GP2).

*The relationship with the local pharmacies is quite an interesting one. Obviously our pharmacy team here know about a lot of tricks that people sometimes pull that aren’t necessarily that ethical, so [the pharmacist] and his team have been challenging them on things that they don’t think are appropriate whereas we just would never have known that it was happening so that’s good. There is sometimes a tendency with local pharmacies to call when they know that the pharmacy team have left so that they get to talk to the doctors instead but we are trying to work our way around that, and say no, you need to call between this time and this time in the morning to speak about any medication queries (GP5).*

#### Trust

There was a pervading sentiment from the participants that they were all working together as a team to advance patient care. Practice staff felt that the pharmacy team had integrated well and cooperated effectively. Pharmacists and technicians reported feeling that they had been accepted into the team with their views valued and contributions appreciated. GPs stated that they trusted the pharmacy team’s ability and this view was reinforced by the pharmacy team. Both parties, however, noted the importance of taking time to build up trust.

*It does take time to build a relationship with anybody in a practice and… I think there is an element of not just his knowledge as a pharmacist but his practical approach to applying that knowledge that makes you trust him more and that probably encourages more positive communication (GP3).*

A few participants suggested that some GPs exhibited initial distrust and a lack of engagement but felt that this was no longer an issue in practices in which they had worked for a while. Pharmacists and two GPs (GP2 & GP3) noted that some GPs might feel threatened by pharmacists.

*I think there would always be a ‘them’ and an ‘us’ in a way but it’s sort of merged a bit more now. I can see a boundary, maybe not this surgery so much, but there’s other surgeries that definitely like to keep a clear role difference. A lot of GPs wouldn’t like to be put on the same level as pharmacists I’ve noticed. Here’s a bit different, there’s been a relationship longer, but a few other surgeries where I've worked for the doctors there’s a bit more tension but I think that will go with time once they’ve worked together alongside each other a bit more (PT4).*

*It’s got to do with personality. It’s the way that GPs work, the pharmacists too. You’ve got to see them as part of the team and not a competitor (GP2).*

One GP compared this perceived threat to professional boundaries and identity to that observed during the introduction of nurse practitioners, although they suggested that this sentiment might be stronger since *everything a nurse can do a GP can probably do, whereas anything a pharmacist can do the GP probably can’t (GP2).* One manager believed that prejudices would be a barrier to project expansion.

*I think its overcoming prejudices of GPs too, allowing someone else to come in and look at their medication budget and prescribing and actually accepting the advice of another clinical professional (M5).*

Initial distrust was not limited to GPs with one pharmacist who visited nursing homes stating that some residential care nurses were very unhappy with their presence and felt that they (the nurses) were being *policed (CP1)* by the pharmacist. One general practice nurse (N3) described her preference to speak to the GP with medication queries despite acknowledging that a pharmacist’s medication knowledge might be superior to that of a GP. Furthermore, another nurse (N6) believed that medication queries were not the role of a pharmacist and that the GP remained the most appropriate contact.

#### Communication

Participant’s views on communication with the pharmacy team within the practice were mixed. Positive experiences included the electronic communication system that enabled rapid responses.

*It’s really helpful using (the internal online messaging system)… I can ask for advice from the pharmacy team and they’re really good at getting back to you. So for me it’s brilliant (N4).*

Some members of the practice staff lacked awareness of the pharmacy team’s working hours raising concerns about short and unfixed hours of work that were a barrier to effective communication. Some receptionists and managers described a failure in communication when the pharmacy team suspended the reauthorisation of prescriptions pending a review but omitted to communicate this to the receptionists.

Practice meetings were the main method of communication with many participants discussing their benefit. The pharmacist’s contribution to education within the practice was identified with many participants mentioning educational talks delivered by the pharmacy team as well as instances of individual advice they received from the pharmacist. GP1, however, questioned why technicians did not regularly attend the practice meetings and then acknowledged that the pharmacist liaised with the rest of the pharmacy team after the meeting.

*Next Monday we have the technicians speaking about the electronic prescribing system to just give reminders because it’s still quite a new process. You know top tips, we try and keep it as open learning as possible for everyone (M7).*

### Practicalities

Practicalities are defined, by the Oxford Dictionary of English, as the aspects of a situation that involve the actual doing or experience of something rather than theories or ideas. A number of the practical issues surrounding pharmacists in general practice were identified including the physical location of the pharmacy team and their hours of employment. The cost of employing a pharmacist was balanced against savings and improvements both for the general practice and the NHS, however, patients’ awareness of the service was poor.

#### Location and space

A strong preference was expressed for the pharmacy team to be located *in house all day* (GP6). In practices where the pharmacy team was located on-site, participants reported easy personal access and the ability to ask informal questions. Where the pharmacy team was located off-site, however, they were viewed as *a separate entity* (GP7) and aspects of communication were lost.

*Having somebody in house, (it) is the corridor talk and it’s difficult to quantify how helpful that is because you can say, “Can I just pick your brains on something?” If he wasn’t here, in the building, I don’t think I would think to ask him. Because he is in the building and because I see him… I do think “Oh actually we’ve got a pharmacist who can look into this” (GP3).*

*(Being located off-site has) been more of a challenge because they (pharmacists) can’t just walk down to my office and say “There’s a problem, can you help me?” Communication wise we have phones, our computer system, we can send instant messages, task, and email so it’s not an issue. Having said that, when you lose face-to-face contact, there could be more misunderstanding and delays (M5).*

One of the challenges most broadly identified was the shortage of accommodation available to house extra members of staff. GP5 & GP6 stated that the lack of accommodation had directly prevented participation for some practices in the national *Clinical Pharmacists in General Practice Pilot* which was due to start.

*The physical problems of the buildings and communication I think are the main issues (M4).*

#### Costs

The cost of employing a pharmacist was seen to be a barrier, however, participants often counterbalanced this opinion against savings generated by the pharmacy team, decreased workloads, decreased prescribing errors and improved patient safety. Participants also described increased efficiency in dealing with the repeat prescribing process with one manager (M1) reporting a reduction in errors and medication-related complaints with more patients being called in for medication reviews.

*Pharmacists in GP surgeries, I don’t really see it as being optional if you consider the patient safety aspect. I think… it can reduce… so many errors (GP3).*

Some participants were also of the opinion that the quality of patient service had improved, with reasons varying from a quicker repeat prescription turnaround time to being able to offer the level of individual service for which GPs did not have time. A nursing home pharmacist (CP1) noted that their role in monitoring patients had led to a noticeable reduction in hospital admissions.

The pharmacy team’s contribution to the reduction in practice drug budgets was widely discussed by a broad spectrum of participants with two GPs directly attributing a 20% drop in the practice prescribing budget to the pharmacy team. GP2 raised the point that although the pharmacy team generated significant savings on drug expenditure for the NHS the cost of employing the team was borne entirely by the practice.

#### Patients’ access

Patients discussed the relative ease of obtaining an appointment with a pharmacist within the week in comparison to 1–3 weeks for a GP appointment. They reported that long waits for GP appointments caused them stress and made them feel like a burden to the NHS. Patients were also very pleased with the duration of their appointment comparing a 30-min pharmacist appointment to 10–15 min with their GP and believed that this allowed for a more beneficial consultation.

*I got this appointment quite quick, so, whereas if it was a doctor I think it was another 2 weeks or something, which, because it was only to review my medication I felt that’s quite a long time to wait just for that, so this is a good way of doing it (PA2).*

*If 4 years ago I had the opportunity to talk to someone like the pharmacist about this situation, it could have been resolved [then]... The hour that I had in two meetings with the pharmacist was more useful than tens of appointments that I had with my GP (PA4).*

Many patients expressed a strong willingness to see the pharmacist again and were happy to book an appointment with the pharmacist rather than the GP often stating that they did not want to bother the doctor with *“silly things”* (PA3) and would rather free up GP appointments for those who might be more deserving.

*All about my medication, he went through each and every one of them and he agreed what I should be on and what I should be told about, he agreed with most of them except for this warfarin. He gave me alternatives and he wrote it down… To get somebody to talk to about my medication when there’s serious people out there that need attending and doctors are giving their time for me to sort my little problem out, you know, whereas if you’ve got somebody like the pharmacist, if you rung up to speak to him and he can sort it out (PA3).*

One patient, however, expressed concerns that as the service becomes more established, appointments will have to be shortened due to increased demand. Another expressed preference for an out-of-hours appointment.

*The cynical view is that once it gets introduced and adopted, because it’s a popular move and if more people find that they need that, so they all pile in and you get one pharmacist here and everybody wants to know a bit more about their drugs. So if they’re given free access to him, he’ll be as busy as the doctor, if not busier (PA5).*

Patients’ awareness of the presence of a pharmacist within the practice was low with some failing to realise that they had seen a pharmacist even when questioned post-consultation, often assuming the interviewers were referring to their community pharmacist.

*I’ve never seen the pharmacist here. Oh, this is the pharmacist? Absolutely excellent, but I’ve seen this gentleman twice… I just made an appointment, I thought, you know I was seeing one of the doctors and I said to him I’ve never seen you before, are you a new doctor, and he said “No, no”, he said “I’m a pharmacist” and he explained to me. I have no idea why they decided, I suppose they decided here, that I was to see a pharmacist because it was more appropriate because I’m old, I take about twelve kinds of medicines (PA7).*

One patient (PA3) felt that the service was under-publicised and expressed concerns that there was no mention of the pharmacist in any communication they received from the practice.

*And you say he’s been here a long time? How come he never appears in any of the paperwork or letters or anything? Because, you know, some letters we get from the surgery and they’ve got all these doctors’ names on it and all that, right? But the pharmacist’s name’s not on it (PA3).*

GP3 suggested that the potential inability of patients to distinguish between a pharmacist and a doctor was a challenge due to differing skillsets. GP6 described being upset that patients do not take the initiative to visit a community pharmacy to treat minor ailments and suggested that *the message doesn’t get through* even though *they can be far better treated by a pharmacist* who is *fantastic at referring people.*

## Discussion

Our findings relate to the experiences of the key stakeholders in the Ealing GP Federation where pharmacy services have been provided for several years. The model used by this group of practices is possibly unique in employing pharmacy technicians to undertake repeat prescribing duties but findings could still be transferable to other sites and models nationally and internationally. Experiences were generally positive and included appreciation of pharmacists’ knowledge of medicines, educational contribution to the practice and their methodical approach to tasks. Receptionists reported increased efficiency, decreased workload, decreased medicines related complaints and a safer repeat prescription process under the direction of a pharmacist. GPs spoke of decreased workloads and more time for patients, resulting in improved relationships. Pharmacists talked about increased job satisfaction and a strong understanding of competencies and boundaries with some requesting further training. There were, however, a few areas that could be improved, including role definition, communication processes, patients’ awareness of services, co-location of pharmacists and general practice staff, and full-time access to pharmaceutical services.

Mossialos et al. [19] reported a lack of policy-relevant evidence to support the expansion of the traditional role of community pharmacists and suggested that the adoption of patient-centred responsibilities might be justified by challenges to the health system such as workforce shortages, an ageing population and increased demand for services. Ours is the first research of its kind in the UK. It addresses the expansion of pharmacists’ roles into general practice and reports findings that pre-empt any to come from pilot sites because of the pre-existing nature of the services studied. In essence, it provides a snapshot into what the future might look like and how the pilot sites could evolve in time. It also enables identification of avoidable pitfalls. Our study provides some insight into the employment of pharmacists in general practice that can feed into future formal evaluations and inform policy development. Our research is also unique in the inclusion of all stakeholders, including patients, in the general practice environment. This allowed for a broad range of experiences from a variety of participants, enabling the findings to be extrapolated to similar general practice situations. Other strengths include the semi-structured interview technique which enabled participants to take the lead in interviews to discuss issues of importance to them. Rigour was ensured by the use of multiple researchers (interviewers and analysts), peer debriefing regarding interviews and developing analytical understandings and regular group meetings to develop consensus on codes, categories and themes.

Limitations include the small number of participants in some stakeholder groups, especially the patients, and the brevity of some interviews due to time constraints of the participants. This work needs to be repeated with larger groups from a variety of practices nationwide or in practices that use very different models of pharmacists’ involvement.

### Role development within multi-disciplinary teams

There is a growing body of literature, mostly from overseas, about the integration of pharmacists into primary care teams [11, 12, 20, 21]. Much of the reported work focuses on professional role development and identity within multi-disciplinary teams and mirrors many of the findings of our study around role definition and inter-professional understanding, expectations and responsibilities. Farrell et al., as part of their IMPACT (Integrating Family Medicine and Pharmacy to Advance Primary Care Therapeutics) project in Ontario, Canada, implemented a mentorship scheme to reduce the stress of adapting to new roles and to promote professional development [22, 23]. In 2013, they produced guidelines for the integration of pharmacists into primary care teams with practical recommendations similar to ours. We suggest that there might be valuable lessons from this work for UK health professionals involved in the integration of pharmacists into general practice, particularly the demonstration of leadership and vision, and the development of practice support networks. Luetsch and Rowett [24] showed in Australia that inter-professional communication skills training “enhanced professional identity, credibility and the ability to build a collaborative working relationship with other health professionals” and improved role satisfaction for pharmacists. Some of the participants in our study hinted at professional boundary and identity issues with comments about ‘knowing limitations’ and ‘deskilling’. Inter- and intra-disciplinary boundaries (between and within professions) have been identified by many researchers as barriers to integrated patient care delivery by multi-disciplinary teams [25–28]. Collaboration is affected by “attempts to defend existing boundaries, specialised knowledge, and pre-existent practices” (Liberati p 32) and, we would add, discipline-specific professional identities. Bardet et al. [29] identified the key elements to initiating collaborative practices as “trust, interdependence, perceptions and expectations about the other healthcare professionals, skills, interest for collaborative practice, role definition and communication” (p612). According to Liberati [26], evidence of how multi-disciplinary teams develop and function, including the social factors that influence knowledge and practice integration is still limited (p32), and, if issues of professional boundaries and identities are not addressed, healthcare practitioners will fall back on their formal roles rather than developing new ways of working together (p38).

Given that we have reported in our study that it takes time to build trusting professional relationships, we suggest that the strengthening of inter-professional communication skills training at both undergraduate and postgraduate levels for all multidisciplinary team members followed up with mentorship in general practice could help break down initial barriers and trust issues more quickly. Since our research was conducted, the Centre for Pharmacy Postgraduate Education (CPPE – funded by Health Education England and hosted by The University of Manchester) has developed a range of clinical programmes and study days for pharmacists and pharmacy technicians. Similarly, the Royal College of General Practitioners (RCGP) has endorsed localised general practitioner/pharmacist/patient joint-working strategies. While we applaud these initiatives we suggest that more could be done around inter-professional education involving all members of general practice teams and including patients.

More recently (2016) the Royal Pharmaceutical Society has produced *The Ultimate Guide for Pharmacists Working in GP Practices.* It includes person specifications and job descriptions, business cases, practical guidance (for example indemnity and insurance, using IT systems and consultation skills), relevant legal and regulatory frameworks, managing and optimising medicines, and professional and clinical guidance [30]. This resource together with our findings and recommendations below provide useful guidance and insight for pharmacists and general practices venturing into this type of partnership.

### Patients’ awareness of pharmacy services

There is very little contemporary knowledge and qualitative data on patients’ awareness and views of receiving pharmaceutical services in general practice in the UK and even less on their information needs. Limited evidence, including from this study, suggests a lack of public awareness about the level of expertise of pharmacists and the range of services already provided [5, 31, 32]. NHS, Department of Health and RPS policy statements and strategic directives over the past decade have all focussed on building on the strengths of pharmacy and delivering improvements in and extension of pharmaceutical services. Thus there is a mismatch between the thrust of policy development and strategic directives [33, 34], service design and delivery [2] and public understanding. *Pharmacy in England*: *Building on strengths – delivering the future* (2008) called for wider public understanding of the breadth of services, including a mass communications programme and mapping of target audiences. We suggest that this work is sorely needed and long overdue.

### Recommendations

The following recommendations are made:

- Develop clear definitions of roles and basic duties, even if they are flexible enough to allow for further development, and provide induction and training needs analyses for new staff members and constant role reminders at regular meetings of all staff members
- Allow time for multi-disciplinary team building, including with surrounding community pharmacists, and the development of trust and leadership
- Establish reliable means of communication – email, telephone, electronic management systems – that include documentation of activities and decisions
- Ideally, provide full-time, on-site pharmacy services or at least regular hours with strategies to prevent deskilling of other health professionals
- Work with patients on their information needs about the pharmacy services provided and the most appropriate health professional to consult.

Further research is required on the different models of incorporating pharmacists, technicians and pharmacy services into general practice. Work is needed on pharmacy intra-professional boundaries, especially the relationships between community pharmacy and general practice personnel. Further work is also required with patients about their preferences for receiving these services, their concerns and information needs.

## Conclusions

Although the integration of pharmacists into general practice is not entirely new there are no recently published UK evaluations of the services provided or the various models in existence. The *NHS England Clinical Pharmacists in General Practice Pilot* is currently being implemented (and further rolled-out) so while we await evaluations, projects like the one reported here will be crucial in providing guidance for integration of pharmaceutical services into primary care, service improvement and expansion, future policy development and the education of health practitioners. In addition, if patients are to benefit from collaborations like this, they need to be involved in service design and public education. Furthermore, general practitioners will need to be vocal about the advantages and their support for pharmaceutical service inclusion in the general practice team.

## Additional file

Additional file 1: Interview guides including indicative questions for pharmacists, general practitioners, managers, nurses, receptionists and patients. (DOCX 16 kb)
