# Supplementary material for: Pharmacists in general practice: a qualitative interview case study of stakeholders’ experiences in a West London GP federation
Source: BMC Health Serv Res. 2018 Apr 2;18:234. doi: 10.1186/s12913-018-3056-3 (PMC5879559; doi:10.1186/s12913-018-3056-3)
Supplement: Supplementary file 1 — Interview guides including indicative questions for pharmacists, general practitioners, managers, nurses, receptionists and patients. (DOCX 16 kb) [file 12913_2018_3056_MOESM1_ESM.docx]

**Additional File 1: Interview Guides**

**Indicative Interview Questions**

**Questions for pharmacists:**

Thank you for taking the time to contribute to our research project.

1. What are your roles and responsibilities within this surgery?

- The role of the pharmacist
- Skills and qualities
- Training
- Patient care
- Day-to-day working life

1. Please tell us about your experiences of working in this clinic

- Relationships with other stakeholders
- Pros and cons
- Impact

1. Overall view on working in surgery

- Feelings
- Initial thoughts and how they’ve changed
- Facilitators and barriers

**Questions for general practitioners:**

Thank you for taking the time to contribute to our research project.

1. Please tell us about your experiences of working with practice-based pharmacists here at this surgery.

- Your own roles and responsibilities – any changes
- Work practice - time, workload, changes
- Relationships

2. How have pharmacists in GP surgeries affected the level of healthcare provided?

- - Impact on prescribing, errors
  - Number of patients seen a day
  - New services

1. Overall view on working in surgery

- Feelings
- Initial thoughts and how they’ve changed
- Facilitators and barriers

**Questions for managers:**

Thank you for taking the time to contribute to our research project.

1. Please tell us about your experience of having a pharmacist in the GP surgery

- Relationships
- Services provided
- Change in work practices
- Advantages and disadvantages

2. How have pharmacists in GP surgeries affected the provision healthcare?

- Number of errors
- Number of patients seen a day
- New services
- Communication
- Teamwork

3. If another GP manager was to seek your advice on hiring a pharmacist in the clinic, what would you tell them?

- Benefits
- Things to consider
- Short term/ long term implications

**Questions for nurses:**

Thank you for taking the time to contribute to our research project.

1. Please tell us about your experience of having a pharmacist working in this clinic

- Roles – changes
- Work practices – time , workload, changes
- Relationships – communication, collaboration
- Responsibilities

2. How have pharmacists in GP surgeries affected the level of healthcare provided?

- Number of errors
- Number of patients seen a day
- New services

3. Overall view on working in surgery

- Feelings
- Initial thoughts and how they’ve changed
- Facilitators and barriers

**Questions for receptionists:**

Thank you for taking the time to contribute to our research project. Please tell us about your experience of working with pharmacists within GP clinics?

1. Please tell us about your experience of having a pharmacist working in this clinic

- Roles – changes
- Work practices – time , workload, changes
- Relationships – communication, collaboration
- Responsibilities

1. How have pharmacists in GP surgeries affected the level of healthcare provided?
   - Number of errors
   - Number of patients seen a day
   - New services
2. Overall view on working in surgery

- Feelings
- Initial thoughts and how they’ve changed
- Facilitators and barriers

**Questions for patients:**

Thank you for taking the time to contribute to our study and for allowing us to conduct an interview with you.

1. Please tell us about your experience of having a pharmacist working in this GP clinic.

- Services
- Relationship - pharmacist, doctor, other practice staff
- Differences between experiences with a community pharmacist and experiences with a pharmacist within the GP clinic.

1. How have pharmacists in GP surgeries affected the level of healthcare provided?

- Waiting times, length of appointments.
- Queries directed to the pharmacist rather than your GP
- Understanding, knowledge and management of your medicines.
- Impact on medication or quality of care, how you take your medicines (compliance)
